# Frequency and Time Domain Analysis of Foetal Heart Rate Variability with Traditional Indexes: A Critical Survey

**DOI:** 10.1155/2016/9585431

**Published:** 2016-04-18

**Authors:** Maria Romano, Luigi Iuppariello, Alfonso Maria Ponsiglione, Giovanni Improta, Paolo Bifulco, Mario Cesarelli

**Affiliations:** ^1^DMSC, University “Magna Graecia”, Catanzaro, Italy; ^2^DIETI, University of Naples “Federico II”, Naples, Italy; ^3^Department of Public Health, University of Naples “Federico II” Hospital, Naples, Italy

## Abstract

Monitoring of foetal heart rate and its variability (FHRV) covers an important role in assessing health of foetus. Many analysis methods have been used to get quantitative measures of FHRV. FHRV has been studied in time and in frequency domain and interesting clinical results have been obtained. Nevertheless, a standardized definition of FHRV and a precise methodology to be used for its evaluation are lacking. We carried out a literature overview about both frequency domain analysis (FDA) and time domain analysis (TDA). Then, by using simulated FHR signals, we defined the methodology for FDA. Further, employing more than 400 real FHR signals, we analysed some of the most common indexes, Short Term Variability for TDA and power content of the spectrum bands and sympathovagal balance for FDA, and evaluated their ranges of values, which in many cases are a novelty. Finally, we verified the relationship between these indexes and two important parameters: week of gestation, indicator of foetal growth, and foetal state, classified as active or at rest. Our results indicate that, according to literature, it is necessary to standardize the procedure for FHRV evaluation and to consider week of gestation and foetal state before FHR analysis.

## 1. Introduction

Foetal heart rate (FHR) monitoring is of great importance to obtain information about foetal health during pregnancy and labour. In particular, electronic monitoring of the FHR (EFM), commonly named cardiotocography when an external Doppler probe is used, is the most employed method to detect foetal distress and prevent neurologic damage or even foetal death [1, 2]. However, despite its usefulness for obstetricians, the problem of EFM is its poor predictive value. It often lacks specificity leading to unnecessary interventions that increase caesarean delivery and operative vaginal delivery rates [1–4]. Moreover, there is often disagreement between obstetricians analysing FHR traces, since the interpretation is usually performed by means of a visual inspection, which obviously lacks objectivity and reproducibility [5, 6]. In recent years, hence, interest has grown in how to recognize changes in FHR that might predict more accurately foetal distress. For example, in order to overcome the subjective nature of FHR interpretation, several attempts have been made to automate the diagnosis of the foetal status and many computerised algorithms have been developed to assess FHR parameters [1, 6–10].

Independently of the recording methods, the main FHR morphologic characteristics and parameters observed by physicians for foetal health evaluation are FHR mean value (which is related to week of gestation), baseline of the FHR, acceleration's rate and shape, deceleration's rate and shape, and FHR variability (FHRV) [11–15]. Among these, FHRV is probably the most important one, since it reflects the activity of autonomic nervous system (ANS) in the foetus who is growing and developing [1, 16–19], although the exact contributions of the two branches of the ANS are still object of investigation even in adult subjects [20]. The study of FHRV, often referred to as the beat-to-beat fluctuations of the FHR signal, could be, like for heart rate variability (HRV) of adult subjects, a base for a more powerful, detailed, and objective FHR analysis and for better knowledge of ANS reactions and its development [11, 16, 21–24]. With respect to the first studies [25, 26], nowadays, the knowledge of FHRV is improved, so that different ranges of variability can be identified in order to classify FHR recordings [27] and its assessment employed to evaluate foetal reactivity and wellbeing in nonstress condition [28]. Even though the presence of good variability may not always be, by itself, a certain sign of reassuring FHR signal (corresponding to a well-oxygenated foetus), most clinicians agree that minimal or absent variability could be an indicator of foetal distress [28–30].

Due to its recognized importance, a large number of new analysis methods have been enforced to obtain more objective and quantitative measures of FHRV [31, 32]. Traditionally, as HRV of adult subjects, FHRV can be studied both in time (statistical indexes) and in frequency (spectral indexes) domain. In the time domain, Short Term Variability (STV) indexes and Long Term Variability (LTV) indexes are usually distinguished. In the frequency domain, different methods have been employed to estimate the power spectral density (PSD), which is widely considered the index that best covers all the information of the heart rate series. However, since FHRV signal shows a nonstationary behaviour, the time-frequency analysis of the FHRV is generally employed [1, 22, 33–39]. As far as time domain analysis (TDA) is concerned, it could present some limitations because it mainly relies on statistical measurements, so it can only describe the magnitude of the variability around an average value, without providing further information about the physiological mechanisms involved [40, 41]. Some limitations have been also shown for the frequency domain analysis (FDA) since it is generally sensitive to artifacts [42] and can provide information only about periodical fluctuations of the heart rate rhythm, without inspecting other possible nonperiodic trends embedded in the variability signal [40, 41]. In order to overcome these limitations and also to investigate and improve risk stratification, during the last decades, techniques analysing nonlinear dynamics, such as symbolic dynamics, approximate entropy, and fractal analysis, have been employed both in adults and in foetuses, even if, at the moment, none appears to be predominant and completely satisfying [26, 43, 44].

TDA and FDA hence remain the most used methods of HRV and FHRV analysis also due to their simplicity and higher acceptance in clinical environments. Nevertheless, a standardized definition of FHRV is still lacking and subject to changes and updates. Traditional analysis lack of standardized methods for computing time or frequency domain indexes and the relationship between FHRV and foetal growth is actually not completely clear and most of the studies inspecting this issue are by now rather old [52, 54, 71, 72].

This works aims to compare some common indexes of TDA and FDA, employing both real and artificial FHR signals.

To get this objective, we firstly introduce a brief report on the most relevant literature works about traditional FHRV analysis, focusing the attention on those studies based on the computation of the STV, as a time domain index, and on the power of the FHRV in different frequency bands.

Then, through the use of simulated FHR signals, we define the methodology to be employed for the estimation of the chosen indexes for FDA.

Finally, we apply the time and frequency indexes to the analysis of real FHR recordings in order to assess the capability of these indexes to correlate with foetal development during gestation and with foetal state and to provide an overview of their reference values.

## 2. Literature Report

### 2.1. Spectral Analysis of FHRV: A Brief Literature Report

The study of biologic signals in the frequency domain can offer deeper knowledge of their behaviour. In adults and foetuses, the spectral analysis permits estimating the power of the periodic HR fluctuations and it represents a noninvasive and powerful tool to understand ANS functional state and reactions [28].

After the study of Akselrod et al. [73], which introduced the power spectral analysis of short term heart rate fluctuations as a noninvasive quantitative probe of beat-to-beat cardiovascular autonomic control, FDA has been widely performed to point out the relation between the ANS activity and low frequency and high frequency bands, whose power content reflects changes of sympathetic and vagal activity [24, 26, 61]. Besides, changes in power distribution have been recognized as predictors of foetal distress, both in antepartum and in intrapartum periods [65, 74]. The work of Padhye et al. [19] investigated the correlation of power in LF (computed using the Lomb periodogram) and HF bands and noted an increasing trend of the power with gestational age. In their study, van Laar et al. [63] used fast Fourier transform to calculate the FHRV spectral power in LF and HF bands in order to compare spectral values between near term and postterm foetuses and found a sympathetic predominance in foetuses near term during active state, but an increased vagal modulation in postterm foetuses during rest state. According to Kwon et al. [4], changes in spectral power corresponding to a low pH are different between term and preterm foetuses, confirming a correlation between frequency indexes and gestational age.

Despite these interesting and important clinical results, problems in interpretation and comparison arise because literature works employ different frequency analysis methodologies, use discordant measure units for the PSD, and show disagreement about the frequency bands of the FHRV spectrum [36, 75], even if most of the literature agrees that, like the case for adult subjects, three bands can be detected in the FHR power spectrum: a very low frequency (VLF)band, which seems to be related to thermoregulation mechanisms [12, 67]; a low frequency (LF) band, which is mainly associated with the sympathetic branch activity and is an indicator of foetal development and wellbeing [54, 67]; a high frequency (HF) band, which reflects the respiratory activity and the vagal stimulation [38, 45, 47, 49, 61].

In order to clarify the definition of frequencies bands, a study of literature was conducted, involving about eight hundred literature works concerning foetal monitoring, published between 1983 and 2013. Among these, only a hundred works are directly related to frequency analysis and only about thirty works gave details about the three bands; the major disagreement is about the VLF band. Some researchers identifies the VLF band in the range from 0 to 0.03 Hz, while others consider VLF band ranging from 0 to 0.04 Hz or from 0 to 0.05 Hz [12, 50, 56, 67]. Furthermore, some authors introduce a middle frequency (MF) band in the range of 0.15–0.5 Hz or 0.2–0.4 Hz [36, 43].

A concise overview of different literature works focused on the computation of the FHRV spectral bands and corresponding power content is shown, respectively, in Tables 1 and 2. Empty cells are due to the absence of data in the original papers.

### 2.2. STV in Foetal Monitoring: A Brief Literature Report

As mentioned, in the time domain, Short Term Variability (STV) indexes and Long Term Variability (LTV) indexes are usually distinguished. The former, also according to FIGO guidelines [76], refer to the continuous small changes in difference between successive interbeat intervals, which occur under physiological conditions. These minimal oscillations cannot be reliably interpreted by the naked eye; furthermore, a correspondent shared mathematical definition is lacking, so that this important parameter has lost part of its relevance and in some more recent guidelines it is not even considered but they speak broadly of variability, referring implicitly to the amplitude of FHR signal and without differentiating by LTV, since in practice they are visually determined as a unit [14, 77, 78].

When a computerised system is available, different indexes are used, many of which are borrowed from studies concerning adult heart rate. Among them there are Root Mean Square Successive Difference (RMSSD), that is, the square root of the mean squared differences of successive RR intervals, and pNN50, that is, the percentage of differences between following RR intervals greater than 50 ms [26, 79]. The LTV, instead, refer to fluctuations in the FHR over seconds, such as SDNN-Index, that is, the mean of the 5-minute standard deviation of the NN interval (normal to normal interval) calculated over 24 h, and SDANN, that is, the standard deviation of the average NN interval calculated over short periods [26].

In clinical practice, beat-to-beat indexes are often preferred since a good beat-to-beat variability is widely accepted as a significant index to assess foetal wellbeing, since a good beat-to-beat variability is a reliable indicator of a healthy foetal ANS [80]. Hence, many studies in time domain attempted to compute indexes for quantifying STV in foetuses by using very different techniques and methods (modification of the mean, standard deviation (SD), slope changes, and varying epoch lengths) [81]. The lack of a unique standardized methodology along with the fact that STV formulas, usually based on ECG, are often applied without any adaptation to the ultrasound technique makes the comprehension of the measure and the comparison between two or more indexes very difficult [79].

Despite the lack of standardization, STV is broadly employed. For example, Short Term Variability was found to be a good predictor of Apgar scores by Ayres-De-Campos et al. [82]. D'Elia et al. [83] analysed healthy term foetuses subjected to vibroacoustic stimulation by means of computerised CTG and found a statistically significant increase in foetal movements, acceleration rates, and STV with foetal activity. In their work, Serra et al. [84] examined the clinical value of the STV in the timing of the delivery of severely growth-retarded foetuses and confirmed that the STV can assess the condition of foetuses with severe intrauterine growth restriction (IUGR) and that it is an important marker of perinatal outcome in severely growth-retarded foetuses. Also Galazios et al. [85] have rather recently observed that STV value is associated with foetal distress and, more recently, the study of Annunziata et al. [86] evaluated the impact of vibroacoustic stimulation on STV of CTG recordings in low and high risk pregnancies and noted that an increase in STV is significantly associated with good perinatal outcome.

Finally, Cesarelli et al. [79] proposed a comprehensive study on nine different mathematical indexes utilised to compute STV from CTG recordings, testing their robustness, sensitivity, and dependence on other parameters (FHR storage rate, FHR mean value, etc.) and demonstrated that the SD index, computed after floating line extraction, provides efficient information and is independent of the considered variables.

## 3. Methods

### 3.1. Data Collection

Real CTG traces were recorded by healthy pregnant women during the clinical practice, using commercially available cardiotocographs (HP-135x or Sonicaid). Five hundred and eighty recordings, lasting on average more than 25 minutes, recorded from women between the 24th and the 42nd gestation weeks, were considered for the study. Gestational age was determined from the last menstruation date or from ultrasound measurements executed in the first trimester of pregnancy. The database was completed with other pieces of clinical information of patients and newborns. All patients gave their informed consent to participate in the research concerning foetal monitoring.

CTG signals were processed by software previously developed by the authors [7, 84]. Firstly, they were preprocessed by means of a software [10, 84] which processes signals in output from the cardiotocographs in order to recognize signal tracts having good and bad quality (these last including tracts of signal loss); for each segment of good quality, recover the real uneven FHR series when CTG output is evenly spaced (case of HP/Philips cardiotocographs) [87] and detect and process outliers [88]; interpolate signal tracts of poor quality (according to an index provided by the equipment) or signal loss which last maximum 3 s, in order to avoid an excessive fragmentation of the signal.

In this way, all CTG signals available for further processing have the same characteristics and are unevenly sampled (in correspondence with the real heart beat) regardless of the equipment employed for their recording, hence regardless of the acquisition and sampling mode.

### 3.2. CTG Simulation

Like it is known, real FHR signals are affected by a considerable amount of variability and complexity, because of relationships, complicated and not yet fully known, among the different physiological mechanisms involved in heart rate regulation. Hence, in order to have available signals with characteristics known a priori, artificial CTG signals were employed for the analysis carried out in order to define an adequate methodology for FHRV evaluation.

**Figure 1:**
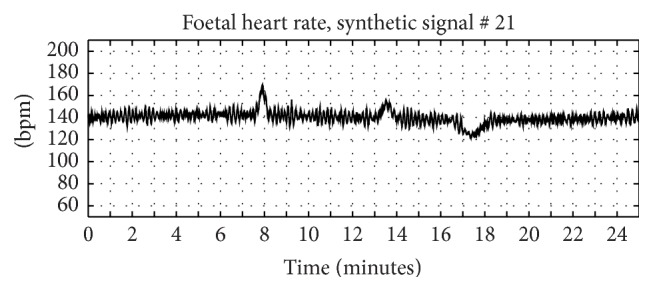
Synthetic FHR. Example of FHR artificially generated according to a procedure previously developed and published.

According to previous works [79, 89, 90], an artificial uneven RR series with specific power spectrum characteristics, proper for FHR, was firstly generated. In particular, we set central frequencies of the spectral bands, the bandwidths, and the ratio between power of low and high frequency bands. Then, in line with the operations made by the cardiotocographs which detect heart beats (by means of Doppler technique), compute interbeats distances series, and then reversing it get the FHR signal, this last one was computed as FHR = 60/RR, fixing FHR mean value and standard deviation of its peak-to-peak amplitude. After that, acceleration rates and deceleration rates with different amplitude and duration were simulated by using Gaussian-like signal tracts (for a more detailed explanation about how these synthetic signals were generated, please refer to previous works of the authors [79, 89, 90] and see Figure 1 for an example).

Our final simulated signals, hence, are in principle similar to those obtained with ultrasound technique [90].

The software for generating artificial CTG signals, as well as that for signal processing employed for his work, was developed using MATLAB R2011a [10].

### 3.3. FHRV Estimation

Previously, we proposed definition of FHRV as difference between FHR signal and its floating line [10, 12]. Of course, for FHRV assessment, the floating line has to be correctly estimated, so that we proposed also a procedure, using spline nonlinear filtering, developed to this aim (the methodology has been recently updated, and results are submitted but not yet published).

Here, as further test, we compared the mean frequency spectrum computed on 30 simulated FHRV signals obtained with two different methodologies: by means of the application of our procedure for floating line estimation or simply as a result of the detrend operation, often employed in the literature [16, 55, 59, 63, 67]. (Let us remember that detrend is an operation which removes the linear trend from a signal; in Matlab it is a default function.)

### 3.4. Frequency Domain Indexes

We estimated FHRV as explained in Section 3.3 and considered for this signal LF (0.05–0.2 Hz) and HF bands (0.2–1 Hz); then, as VLF band, we computed the power spectral density (PSD) of the floating line.

Because of the nonstationarity of FHR and hence of FHRV, PSD was estimated by means of the Short Time Fourier Transformation [10], the methodology still more used for its simplicity, using a sliding Hamming window of 32 s [34, 47]. This window is shifted sample by sample and a new PSD is computed each time [35]. To be able to use the STFT, the FHR signal was previously interpolated (4 Hz sampling rate) by means of cubic interpolation, which has been demonstrated to reduce the error introduced [37]. Then, by means of a simple integral rule, we have computed the power values, absolute (called *P*_LF_, *P*_HF_), and percentage, with respect to the total power (called LF%, HF%), in the bands defined above. Further, the sympathovagal balance (SVB) index, which is an important index that reflects the relations between vagal and sympathetic branches of the ANS [26], was computed as ratio between *P*_LF_ and *P*_HF_. Finally, using the same methodology, we computed *P*_VLF_ (and VLF%) as power of PSD of the floating line (using a commercial personal computer with a processor i5-3337U@1.8 GHz and RAM of 8 GB, the computation requires about 4 s for a CTG signal of more than 2 hours of length and about 10 s when also a 3D representation of the PSD is required).

### 3.5. Time Domain Indexes

As index of variability in time domain, we chose STV for its importance in monitoring of foetal health, since it is related to regulation mechanisms elicited by ANS activity [84, 86], as already mentioned in the previous sections. In agreement with a previous study of the authors, we assessed STV as standard deviation of the FHRV, obtained after subtraction of the floating line from the FHR signal [79]. In order to enrich the available information, without worsening the computational complexity, the calculation is carried out on sliding windows of length *M* (with *M* covering 30 s), with an overlap of *M* − 1 samples [10]; then, to provide an overall STV index of the signal, the mean of all STV values is computed (the computation and the graphical representation require about 8 s for a CTG signal of more than 2 hours of length, using the same personal computer utilised for FDA).

### 3.6. Week of Gestation and Foetal State

To test time and frequency indexes, we chose to verify their relationship with the week of gestation, the most simple indicator of foetal growth, and the foetal state.

It is known that foetal behavioural states have a great importance in influencing FHR patterns. However, the exact recognition of the foetal state is not a simple task, since respiratory acts, eyes closure and opening, just to name some aspect, should be simultaneously detected by ultrasound imaging. Hence, according to the literature [36, 63], for CTG recorded from 30th week of gestation onward, we defined active or resting foetal state. In particular, we classified a FHR signal as recorded from a foetus in active state if, at visual inspection, it showed a good variability (at least equal to 5 bpm) and at least two acceleration rates in 20 minutes of recording. If the signal showed low variability and absence of acceleration rates, the foetus was classified as at rest. Doubt cases were excluded from the analysis.

**Figure 2:**
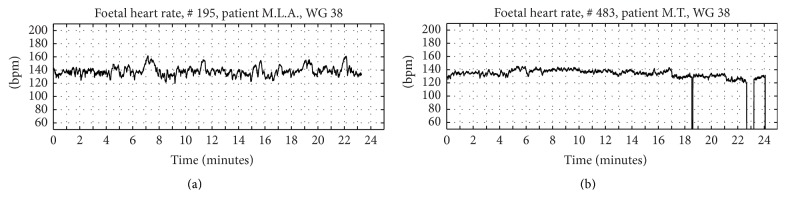
Foetal states. Example of FHR recorded from a foetus in an active state (a) and a foetus at rest (b) WG: week of gestation.

In Figure 2, examples of FHR signals classified as active and rest are shown ((a) and (b), resp.).

### 3.7. Statistical Analysis

For all indexes studied, we analysed the correlation with week of gestation by means of regression lines (a polynomial quadratic curve was considered).

Besides, we tested the ability of these indexes to differentiate active from rest foetal state by means of the Mann-Whitney test, because of the non-Gaussian distribution of data.

## 4. Results

### 4.1. FHRV Mean Spectrum

**Figure 3:**
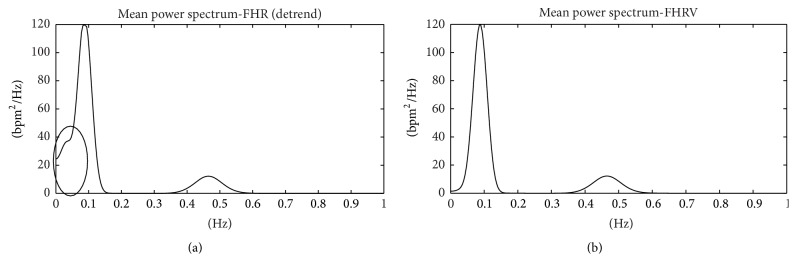
FHRV power spectrum. Mean power spectrum of FHRV computed using detrend (a) and mean power spectrum of FHRV computed after floating line subtraction (b). In the oval (a), the part of VLF band modified by a residual of VLF band.

In order to verify which is the more appropriate operation for FHRV estimation, detrend or floating line subtraction, in Figures 3(a) and 3(b), we show results of the frequency analysis conducted on simulated FHR signals.

It is clear that when the detrend operation is used (Figure 3(a)), a residual of VLF component appears superimposed on LF component. Of course, that can alter power computation and results of sympathovagal balance estimation.

### 4.2. Foetal State

In Table 3, the results of the Mann-Whitney test carried out for all tested indexes are shown; they concern the comparison between foetuses at rest (55 CTG recordings) and active foetuses (384 CTG).

### 4.3. FHRV Indexes

Since all indexes, except SVB, resulted significantly differently in active state with respect to rest state, in Tables 4 and 5 we report ranges (minimum and maximum value) of all indexes here analysed and their mean and SD, computed on real FHR, separately for the two foetal states.

### 4.4. Foetal Development

**Figure 4:**
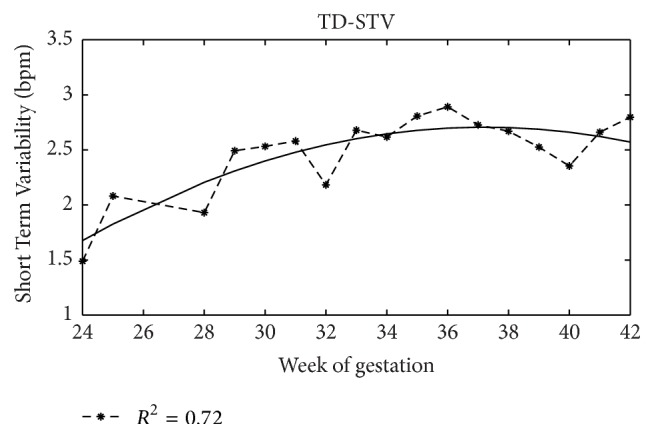
STV trend. Short Term Variability values and regression curve with week of gestation.

**Figure 5:**
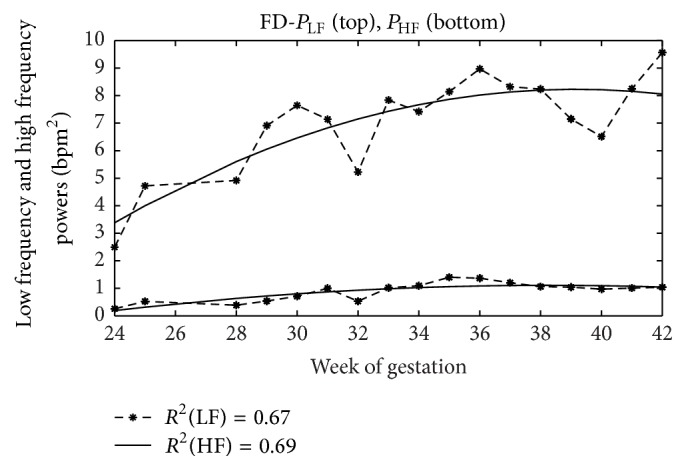
*P*_LF_ and *P*_HF_ trends. Power values in LF (on the top) and HF (on the bottom) bands and regression curves with week of gestation.

**Figure 6:**
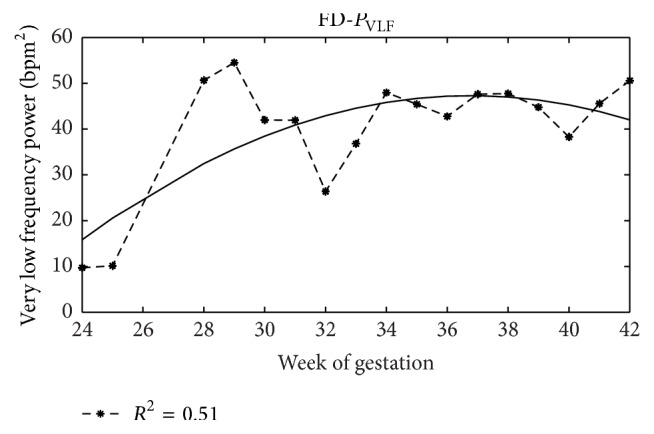
*P*_VLF_ trend. Power values of VLF band and regression curve with week of gestation.

**Figure 7:**
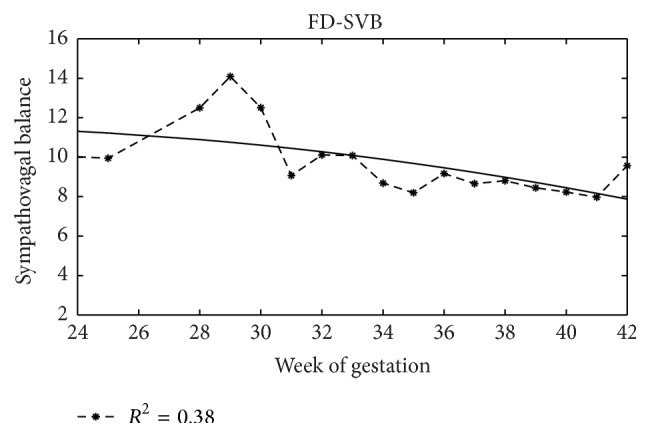
SVB trend. Sympathovagal balance values (dimensionless) and regression curve with week of gestation.

In Figures 4–7, as examples, the trends with the pregnancy course of average values of some analysed indexes are depicted and in Table 6 values of coefficient of determination (*R* ^2^) are shown for all indexes here analysed. In this case, the analysis is carried out without distinguishing foetal states that are not defined early in pregnancy.

## 5. Conclusions

In this paper, firstly we provided an overview of the literature concerning the use of some traditional indexes. It is a concise overview but, at the best of our knowledge, it is the first time that so many quantitative indications and results are compared (Tables 1 and 2).

In the review section, we reported just some clinical results (being this aspect out of the main aim of the work and already treated in the literature [75]) and we neglected the choice of the methodology to compute PSD, since in a previous work we compared three methodologies and obtained not so different results. So, we focused on the definition of FHRV (since a FHRV definition shared and mathematically translatable is still missing) and frequency bands.

As here shown, about the frequency spectrum, although there is agreement in considering three main bands, VLF, LF and HF, and a MF when a more detailed analysis is necessary, even the same author or authors of the same research group employ different bands limits in their works (Table 1). Besides, frequency analysis is often carried out without providing power values and, in case, a large variability is present among different papers (Table 2), so that a comparison is quite difficult.

Furthermore, it is worth underlying that the analyses are very often carried out starting from RR intervals, maybe for historical reasons or for “continuity” with studies involving adult subjects. Nevertheless, ECG is not yet so diffused in clinical routine for difficulty in signal processing (in case of recording through maternal abdomen) or ethical reasons (in case of direct fECG), whereas clinicians are used to analyse FHR signal, measured in beats per minute (bpm).

For these reasons, we prefer to process FHR signal (without converting it into RR signal) and propose definition of FHRV as the difference between FHR and floating line. Preliminary results here shown (Figure 3) confirmed the usefulness of this methodology. About PSD computation, in previous works [34, 37], we used the Lomb method which can be applied directly on uneven series; however, it has too long computational time by losing an advantage of the frequency analysis that can be used, if desired, also for real-time analysis; therefore, for successive as well as for this work we employed STFT. Furthermore, comparing different papers and taking into account experimental results obtained on our database, we decided to define for FHR signals the following bands limits: 0–0.05 Hz for VLF; 0.05–0.2 Hz for LF; and 0.2–1 Hz for HF.

Once defined details of the procedure are to be employed, through a retrospective study on a very large amount of CTG data recorded during physiological pregnancies (439 signals whereas most papers report results computed on a much smaller number of signals), we provided range of values of all frequency indexes here considered (differentiated on the basis of the foetal state, Tables 4 and 5). Some of them show high values of SD that put in evidence the high intersubject variability, even in healthy foetuses.

About STV index, the literature is even in more disagreement and we did not find reference values to report in our brief summary. However, we limited our analysis to a literature overview of the main results obtained in its clinical use since previously we already analysed different mathematical formula employed for its assessment and proposed a new evaluation methodology. By means of this methodology, we computed its value in healthy foetuses and for week of gestation. It, on average, resulted in increase with gestational age (Figure 4), according to the literature [91], and is very different in rest or active foetal state.

Then, we carried out a statistical analysis to test the correlation between the different indexes employed and the foetal growth (week of gestation).

Regression analysis showed that, on average, only some indexes can represent foetal growth in a satisfying way (*R* ^2^ about 0.7, Table 6). Moreover, although a reliable comparison is rather difficult, because of the significant differences in employed methodologies and in a general lack of details provided, as proved here and in the literature [75], they are substantially consistent with others. Absolute LF power and HF power, for example, (Figure 5), increase with gestational age but decrease their growth rate towards the end of pregnancy [58, 61]. With regard to the VLF, we do not have clear literature results with which we make a comparison; however, we may observe that the power in this band increases with week of gestation (Figure 6) and this is coherent with the increase of foetal movements and acceleration rates [61, 92] (let us remember that we computed *P*_VLF_ as power of the floating line which, in turn, includes acceleration rates and deceleration rates). Besides, its value represents the most of the total power so that the two indexes have an equivalent behaviour both with gestational age and with regard to foetal state. We can observe also that in the last weeks of gestation all powers change really little (both in absolute and percentage values), confirming the almost ended development of the SNA (further modifications will concern the adaptation to postnatal life).

The trend of SVB (Figure 7) is an exception but it is not a surprising result. Its value decreases with gestational age, coherently with the literature [36, 56, 61], and with the increase of HF band, related to development of the vagal branch.

Finally, about the foetal state, almost all indexes appeared to be able to separate the two groups (Table 3).

The findings we get in this work lead us to say, in accordance with the literature [63, 75], that standardization of FHRV assessment is necessary and that, since foetal state and gestational age can strongly affect results, it is not possible to process FHR signals regardless of these conditions.

## Figures and Tables

**Table 1:** Literature overview of FHRV spectral bands.

Reference	First author	Year	VLFl	VLFu	LFl	LFu	MFl	MFu	HFl	HFu
[45]	Divon	1985							0.70	0.95
[38]	Cerutti	1989	0.00	0.03	0.04	0.15			0.20	0.40
[46]	Ferrazzi	1989							0.70	0.90
[47]	Karin	1992							0.60	0.80
[48]	Metsälä	1993			0.025	0.069	0.07	0.129	0.13	1.00
[16]	Sibony	1994	0.02	0.05	0.05	0.15			0.15	0.50
[49]	Oppenheimer	1994			0.00	0.20			0.70	0.95
[50]	Groome	1994	0.00	0.04	0.04	0.20			0.20	2.50
[17]	Sibony	1995	0.02	0.04	0.04	0.16			0.16	0.30
[18]	Sibony	1995							0.20	0.50
[51]	Rassi	1995			0.07	0.12			0.56	1.10
[52]	Kimura	1996			0.00	0.30				
[53]	Moczko	1998	0.01	0.10					0.60	1.00
[54]	Ohta	1999			0.06	0.31			0.30	0.50
[55]	Rantonen	2000			0.03	0.07	0.07	0.13	0.13	1
[56]	Zhuravlev	2002		0.05	0.05	0.20			above 0.2	
[57]	Magenes	2002			0.04	0.15	0.15	0.50	0.50	1.00
[43]	Signorini	2003	0.00	0.30	0.03	0.15	0.15	0.50	0.50	1.00
[58]	Van Leeuwen	2003			0.04	0.15			0.15	0.40
[19]	Padhye	2004			0.05	0.25			0.25	1.00
[59]	Yum	2004			0.04	0.15			0.15	0.40
[60]	Siira	2005			0.04	0.15			0.15	1.00
[61]	David	2006	0.01	0.08	0.08	0.20			0.40	1.50
[62]	Tsoulos	2006	0.00	0.03	0.03	0.15	0.15	0.50	0.50	1.50
[36]	David	2007	0.02	0.08	0.08	0.20	0.20	0.40	0.40	1.70
[63]	van Laar	2009			0.04	0.15			0.40	1.50
[64]	Schneider	2009	0.02	0.08	0.08	0.20			0.40	1.70
[4]	Kwon	2012			0.04	0.15	0.15	0.50	0.50	1.00
[65]	Warrick	2012			0.03	0.15	0.15	0.50		
[66]	Reinhard	2012			0.04	0.15			0.15	0.40
[67]	Gonçalves	2013	0.00	0.03	0.03	0.15	0.15	0.50	0.50	1.00
[68]	Van Laar	2013			0.04	0.15			0.40	1.50
[69]	Van Leeuwen	2014			0.08	0.20			0.40	1.70

Results from the studied literature works (listed in chronological order) on FHRV spectral bands (l and u indicate, resp., the lower and upper limit of each band).

**Table 2:** Literature overview of FHRV power values.

Reference	First author	Year	VLF	LF	MF	HF	Measure unit	Foetal state
[38]	Cerutti	1989	27.30		9.30	44.20	%	Breathing
			69.20		25.30	2.60	%	Nonbreathing
			8.10		2.80	10.20	ms^2^	Breathing
			75.40	27.60	2.90		ms^2^	Nonbreathing

[50]	Groome	1994	35.6 ± 15.3	28.6 ± 10.7		35.8 ± 13.2	%	Breathing
			30.9 ± 11.6	28.6 ± 9.5		40.5 ± 13.9	%	Nonbreathing
			0.9 ± 0.67	0.62 ± 0.37		0.77 ± 0.29	ms^2^	Breathing
			0.4 ± 0.43	0.33 ± 0.24		0.42 ± 0.21	ms^2^	Nonbreathing

[43]	Signorini	2003		324 ± 174	28 ± 26		ms^2^	Active
				123 ± 95	16 ± 9		ms^2^	Quiet
			31.10	56.84	8.37	0.18	%	Active
			33.9 ± 15.7	48.3 ± 18.1	12.4 ± 5.6	1.27 ± 1.31	%	Quiet

[59]	Yum	2004		100.5 ± 6.3		15.5 ± 0.9	ms^2^	

[63]	van Laar	2009		0.8 ± 0.08		0.07 ± 0.03	%	Active
				0.69 ± 0.1		0.14 ± 0.06	%	Quiet
				429 ± 410		21.3 ± 7.3	ms^2^	Active
				92 ± 79.9		10.5 ± 5.3	ms^2^	Quiet

[70]	Ferrario	2009	274.82 ± 234.41	136.76 ± 84.21	19.13 ± 10.93	4.8 ± 3.61	ms^2^	
				83.82 ± 4.79	12.26 ± 2.51	3.93 ± 2.55	%	

[69]	Van Leeuwen	2014		45 ± 43		24 ± 12	ms^2^	

Results from the studied literature works (listed in chronological order) on FHRV power estimation.

**Table 3:** Results of the Mann-Whitney test.

		Rest # CTG: 55	Activity # CTG: 384	Mann-Whitney *U*	*p*
		Median value			
DT	STV	1.74	2.80	1850	*∗∗∗∗*

DF	*P*_VLF_	11.3	44.7	1366	*∗∗∗∗*
	*P*_LF_	3.22	7.87	2123	*∗∗∗∗*
	*P*_HF_	0.41	0.99	2526	*∗∗∗∗*
	*P*_tot_	15.8	55.5	958	*∗∗∗∗*
	SVB	8.20	8.54	11124	ns
	VLF%	76.5	83.4	7196	*∗∗∗∗*
	LF%	20.2	14.4	7291	*∗∗∗∗*
	HF%	2.56	1.73	7348	*∗∗∗*

*∗∗∗* for *p* < 0.001 that is statistically highly significant; *∗∗∗∗* for *p* ≤ 0.0001; ns for not significant.

**Table 4:** Values ranges of all indexes.

	STV [bpm]	*P*_VLF_ [bpm^2^]	*P*_LF_ [bpm^2^]	*P*_HF_ [bpm^2^]	*P*_tot_ [bpm^2^]	SVB	VLF%	LF%	HF%
Mean	**1.80**	**14.72**	**3.57**	**0.47**	**18.76**	**8.37**	**74.04**	**22.40**	**3.23**
SD	**0.42**	**10.32**	**1.93**	**0.24**	**10.78**	**3.85**	**14.24**	**12.13**	**2.93**
Minimum	1.13	1.79	0.85	0.16	4.31	1.91	31.10	5.40	0.43
Maximum	2.88	46.46	9.49	1.44	50.89	19.21	94.18	64.87	17.02

Rest foetal state [55 CTG].

**Table 5:** Values ranges of all indexes.

	STV [bpm]	*P*_VLF_ [bpm^2^]	*P*_LF_ [bpm^2^]	*P*_HF_ [bpm^2^]	*P*_tot_[bpm^2^]	SVB	VLF%	LF%	HF%
Mean	**2.90**	**52.39**	**9.04**	**1.24**	**62.50**	**8.53**	**81.31**	**16.19**	**2.41**
SD	**0.78**	**30.12**	**4.64**	**0.94**	**31.36**	**3.62**	**10.06**	**8.31**	**2.28**
Minimum	1.53	11.20	2.05	0.20	15.65	1.92	38.67	2.68	0.26
Maximum	5.77	179.64	27.09	5.61	189.59	20.64	97.06	47.88	16.42

Active foetal state [384 CTG].

**Table 6:** Values of the coefficients of determination (*R* ^2^).

STV	*P*_VLF_	*P*_LF_	*P*_HF_	*P*_tot_	SVB	VLF%	LF%	HF%
0.72	0.51	0.67	0.69	0.57	0.38	0.12	0.16	0.03
